# Facile preparation of core@shell and concentration-gradient spinel particles for Li-ion battery cathode materials

**DOI:** 10.1088/1468-6996/16/1/015006

**Published:** 2015-02-06

**Authors:** Takahiro Kozawa, Makio Naito

**Affiliations:** Joining and Welding Research Institute, Osaka University, 11-1 Mihogaoka, Ibaraki, Osaka 567-0047, Japan

**Keywords:** lithium-ion battery, spinel cathode, mechanical synthesis, core@shell, concentration-gradient

## Abstract

Core@shell and concentration-gradient particles have attracted much attention as improved cathodes for Li-ion batteries (LIBs). However, most of their preparation routes have employed a precisely-controlled co-precipitation method. Here, we report a facile preparation route of core@shell and concentration-gradient spinel particles by dry powder processing. The core@shell particles composed of the MnO_2_ core and the Li(Ni,Mn)_2_O_4_ spinel shell are prepared by mechanical treatment using an attrition-type mill, whereas the concentration-gradient spinel particles with an average composition of LiNi_0.32_Mn_1.68_O_4_ are produced by calcination of their core@shell particles as a precursor. The concentration-gradient LiNi_0.32_Mn_1.68_O_4_ spinel cathode exhibits the high discharge capacity of 135.3 mA h g^−1^, the wide-range plateau at a high voltage of 4.7 V and the cyclability with a capacity retention of 99.4% after 20 cycles. Thus, the facile preparation route of the core@shell and concentration-gradient particles may provide a new opportunity for the discovery and investigation of functional materials as well as for the cathode materials for LIBs.

## 1. Introduction

LIBs have been supporting the development of wide applications from portable electric devices to energy storage systems of renewable energy to build a sustainable society. These offer a high operation voltage and energy density, a low self-discharge rate, flexibility and lightness compared with traditional batteries [1–3]. It is generally accepted that cathode materials for LIBs play a key role in their performance advances [4–6]. Since the discovery of LiCoO_2_ as a cathode material [7], Li-intercalation oxides with transition metals have been found and investigated over the last few decades. Among oxide-type cathodes, LiMn_2_O_4_-based spinel oxides are attractive cathodes for high-power applications such as electric vehicles (EVs), hybrids and plug-in hybrid EVs [8]. In a pure LiMn_2_O_4_ spinel, although it has received attention because of its high-power capability and environmental friendliness [9, 10], the dissolution of Mn^3+^ at a high temperature causes a significant capacity fade [11]. Meanwhile, the Ni-doped LiMn_2_O_4_ spinel, LiNi_0.5_Mn_1.5_O_4_, has a better cycling behavior [12–14]. The highest operation voltage of 4.7 V among the transition metal-doped LiMn_2_O_4_ spinels is also one of the advantages of LiNi_0.5_Mn_1.5_O_4_ [15]. This high operation voltage leads to an increase in the energy density of LIBs. However, the electrochemical and physicochemical properties of cathodes roughly depend on their crystal structures and the transition metal ions, so there is a limitation of the characteristics in the active cathode material of a single component.

Recently, the particle design for cathode materials has become a hot topic in the field of LIBs [16]. In particular, a core@shell structure, which has the expected synergistic effects of a core and shell, has attracted much attention [17]. A significant breakthrough of cathode materials with the core@shell structure has been energetically reported by Sun’s group [18–20]. The first designed core@shell particle was demonstrated in the layered cathode materials and is composed of the Li(Ni_0.8_Co_0.1_Mn_0.1_)O_2_ core with a high capacity and the Li(Ni_0.5_Mn_0.5_)O_2_ shell with an excellent thermal stability [18]. In the spinel-type cathode materials, the particle composed of the Li_1.1_Mn_1.9_O_4_ core and the LiNi_0.5_Mn_1.5_O_4_ shell has been delivered [21]. This core@shell spinel cathode showed an excellent cyclic performance because the LiNi_0.5_Mn_1.5_O_4_ shell protects the dissolution of Mn^3+^ from the Li_1.1_Mn_1.9_O_4_ core. However, a structural mismatch and a difference in volume change between the core and shell encounter the unfavorable problem of void formation at the interface [19, 20], which leads to a diffusion barrier of Li^+^.

To overcome the structural mismatch, concentration-gradient approaches in the shell [22–24] or the entire particle [25–27] have been proposed in terms of the improvement of their performances for the above layered and spinel-type core@shell cathodes. The interfacial barrier between the core and shell can be mitigated by the gradient design, which makes possible the stepwise tuning of material properties. Usually, the preparation of the core@shell and concentration-gradient cathode particles can be attained by a precisely controlled co-precipitation route [18–27]. Although the tailored synthesis of the particles with high quality and a uniform size are successfully conducted via the co-precipitation method, the reaction conditions (pH and concentration of the solutions, temperature, agitation speed, aging time, etc.) should be carefully controlled. For the development and investigation of the core@shell and concentration-gradient cathode materials, the development of a simple and effective method to prepare these particles is of much interest from a viewpoint of the practical applications as well as the fundamental material sciences. Here, we report on a facile preparation route of the core@shell and concentration-gradient spinel particles by dry powder processing. The core@shell particle was prepared by mechanical treatment using an attrition-type mill, and the concentration-gradient spinel particle was produced by calcination of the core@shell particle, which was used as the precursor. The resultant concentration-gradient spinel cathode with an average composition of LiNi_0.32_Mn_1.68_O_4_ exhibited the favorable electrochemical performances. The method presented in this paper provides a new approach to the particle design of cathode materials for LIBs.

## 2. Experimental

### 2.1. Preparation of a MnO_2_@Li(Ni,Mn)_2_O_4_ core@shell and of concentration-gradient spinel particles

Firstly, we prepared MnO_2_@Li(Ni,Mn)_2_O_4_ core@shell particles by a mechanical process. MnO_2_ (>85% purity, Kanto Chemical, Japan) sieved to obtain the particle size of 45–100 *μ*m, Li_2_CO_3_ (>99% purity, Sigma-Aldrich, USA) and NiO (99.8% purity, Sigma-Aldrich, USA) powders were used as the raw materials. These raw materials at a molar ratio of Li:Ni:Mn = 2:1:3 (total amount of their powders, 60 g) were put into the chamber of an attrition-type mill. The milling apparatus has been illustrated elsewhere [28]. Mechanical treatment using the attrition-type mill was conducted for 18 min at room temperature, where the rotation speed of the rotor was controlled below 4500 rpm. The obtained MnO_2_@Li(Ni,Mn)_2_O_4_ core@shell particles were collected and used as the precursor of concentration-gradient spinel particles. Preparation of the concentration-gradient spinel particles was carried out by calcination of the MnO_2_@Li(Ni,Mn)_2_O_4_ core@shell particles at 600–800 °C for 2 h in air.

### 2.2. Characterization

The products were characterized by powder x-ray diffraction (XRD, D2 PHASER, Bruker AXS, Germany) using Cu *Kα* radiation at room temperature. The operating voltage and current were maintained at 30 kV and 10 mA, respectively. The lattice parameters of the concentration-gradient product were calculated by the least square method using silicon (99.999% purity, Sigma-Aldrich, USA) as an internal standard material. The morphology and elemental distribution of the products were observed via scanning electron microscopy-energy dispersive x-ray spectrometry (SEM-EDS, JSM-6010LA, JEOL, Japan). To obtain the cross-sectional image, the powder samples were mounted in carbon and polished to a mirror-like surface. The particle size distribution was determined by the laser diffraction-scattering method (Microtrac MT3300EXII, NIKKISO, Japan). Small amounts of the sample were dispersed in 0.05 wt% sodium hexametaphosphate (Kishida Chemical, Japan) solution using an ultrasonic bath and a homogenizer. Nitrogen adsorption-desorption measurements (3Flex, micromeritics, Japan) were performed to obtain the specific surface area (*S*_w_) and pore size distribution of the products. Prior to each measurement, the powder samples were outgassed under vacuum at 120 °C for 3 h. The specific surface area was calculated by the Brunauer–Emmett–Teller (BET) method in a relative pressure range of 0.12–0.20. The equivalent BET diameter (*d*_BET_) was estimated from the following equation: *d*_BET_ = 6/(*ρ* × *S*_w_), where *ρ* is a theoretical density. For the pore size distribution, the adsorption branch was used in the BJH method. The average chemical composition of the products was analyzed by inductively coupled plasma-atomic emission spectroscopy (ICP-AES, SPS5100, SII nanotechnology, Japan). The samples were dissolved in a mixture of HNO_3_ and H_2_O_2_, and then diluted with ultrapure water.

### 2.3. Electrochemical measurements

The electrochemical performances of the concentration-gradient spinel cathodes were evaluated using CR2032 coin-type half-cells with Li metal as the anode. The prepared concentration-gradient spinel powders were mixed with acetylene black (DENKA, Japan) and polyvinylidene fluoride (Kishida Chemical, Japan) (80:15:5 wt%) in N-methylpyrrolidone (Kishida Chemical, Japan). The obtained homogeneous slurry was coated onto Al foil by the doctor blade method and dried at 100 °C in a vacuum. The dried cathode was uniaxially-pressed and punched out. The coin-type half-cells were assembled in a glovebox filled with dry argon. A polypropylene membrane (Celgard #2400, Celgard, USA) and 1 M LiPF_6_ (Kishida Chemical, Japan) in a mixture of ethylene carbonate and diethyl carbonate (1:1 vol %) were used as a separator and an electrolyte, respectively. The galvanostatic charge and discharge tests were performed on a VMP system (Bio-Logic, France) in a voltage range of 3.0–5.0 V at room temperature. The cyclic voltammetry was conducted in a voltage range of 3.2–5.0 V at a scan rate of 0.2 mV s^−1^.

## 3. Results and discussion

**Figure 1.**
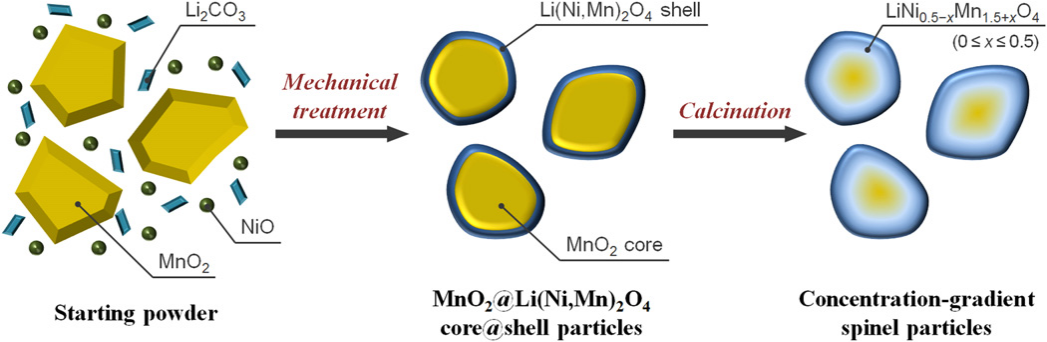
Schematic illustration of the formation process for the core@shell and concentration-gradient spinel particles.

As illustrated in figure 1, the concentration-gradient spinel particles were synthesized via mechanical synthesis of the MnO_2_@Li(Ni,Mn)_2_O_4_ core@shell particles and a following calcination step. At the beginning, the MnO_2_@Li(Ni,Mn)_2_O_4_ core@shell particles as a precursor were prepared by mechanical treatment of the raw materials using an attrition-type mill. Recently, we demonstrated the mechanical synthesis of LiNi_0.5_Mn_1.5_O_4_ using Li_2_CO_3_, NiO and MnO_2_ as raw materials [29]. The formation of LiNi_0.5_Mn_1.5_O_4_ arises from a solid-state reaction at the particle surface of MnO_2_, which is induced by the mechanical stresses and frictional heat applied to a powder layer. If the raw MnO_2_ particles with a micrometer scale are used on the mechanical treatment, the formation of a spinel phase as the shell and the survival of a MnO_2_ phase as the core is predicted. Hence, the MnO_2_ particles with the median size of 43 *μ*m estimated from the particle size distribution were used as the raw material.

**Figure 2.**
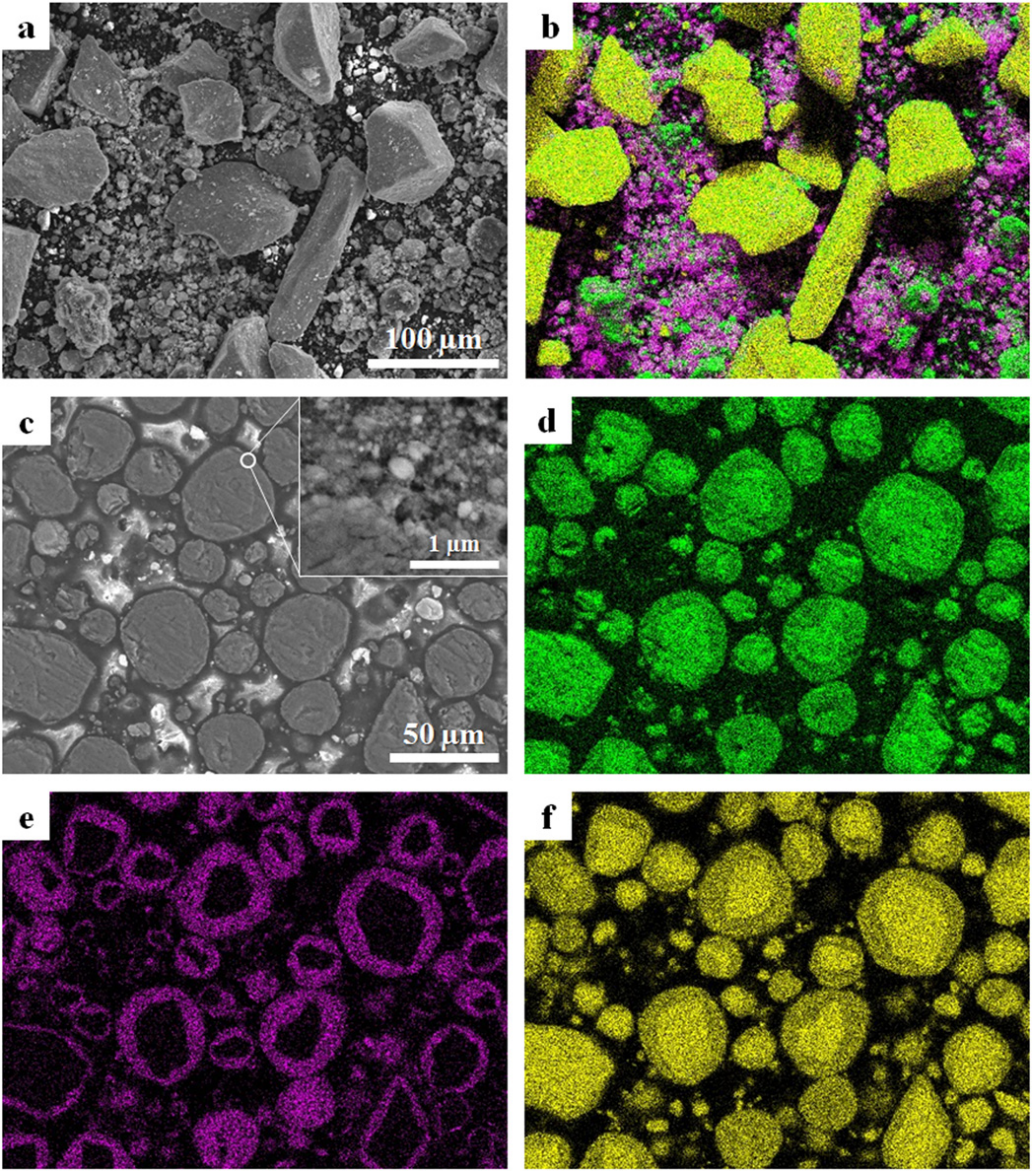
SEM images and EDX elemental maps for the (a), (b) starting powder and (c)–(f) mechanically treated product. Elemental maps of oxygen, nickel and manganese are shown in green, purple and yellow, respectively.

The SEM-EDS elemental maps of the stating powder and the mechanically treated product are shown in figure 2. The mechanically treated product indicates the cross-sectional view of the particles. The starting powder consisted of the micrometer-sized MnO_2_ particles with an irregular shape and the fine particles of Li_2_CO_3_ and NiO (figures 2(a), (b)). The product with a spherical shape was obtained by mechanical treatment of the starting powder using an attrition-type mill (figure 2(c)). A cross-sectional observation of the product revealed the deposition of nanometer-sized particles onto the surface (figure 2(c), inset figure). The EDS maps of oxygen, nickel and manganese clearly exhibited that the mechanically treated product had a core@shell structure and that the shell was constructed by the deposited particles (figures 2(d)–(f)). Nickel was detected in the surface layer of the product particle, while oxygen and manganese were homogeneously distributed. Thus, the core is attributed to a MnO_2_ phase. The XRD pattern of the product, which is shown later, indicated a Ni-doped LiMn_2_O_4_ phase, i.e. a Li(Ni,Mn)_2_O_4_ spinel. Consequently, the MnO_2_@Li(Ni,Mn)_2_O_4_ core@shell particles could be prepared by the simple mechanical process without external heating.

**Figure 3.**
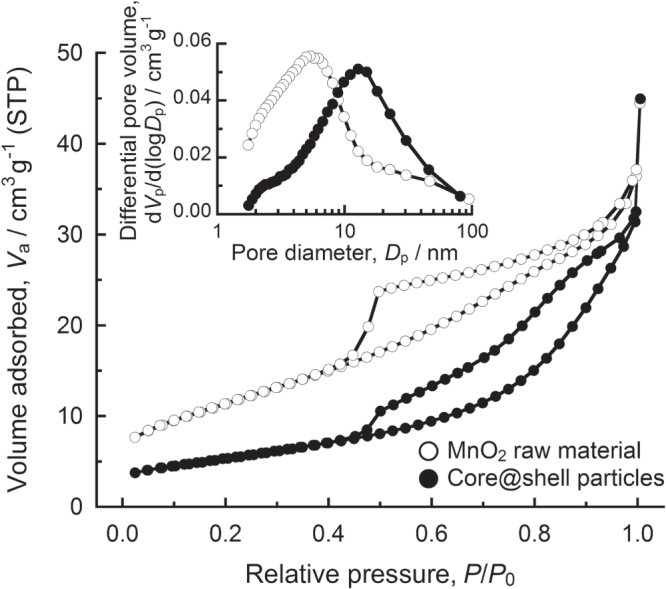
Nitrogen adsorption-desorption isotherms of the MnO_2_ raw material and MnO_2_@Li(Ni,Mn)_2_O_4_ core@shell particles.

The powder properties of the resultant MnO_2_@Li(Ni,Mn)_2_O_4_ core@shell particle were compared with those of the MnO_2_ raw material to discuss the formation of the core@shell particles. The particle size distribution after mechanical treatment shifted to a smaller size. The median size of the core@shell particles was 21 *μ*m and decreased from 43 *μ*m of the MnO_2_ raw particles. The nitrogen adsorption-desorption isotherms shown in figure 3 revealed microstructure of the particles. Both the MnO_2_ and core@shell powders showed a hysteresis loop at a relative pressure from 0.45 to 0.99. According to the pore size distributions calculated from the adsorption branches (figure 3, inset figure), the average pore diameter increased from 5.4 nm to 10.1 nm after mechanical treatment. On the other hand, the specific surface area and the pore volume decreased from 41.1 m^2^ g^−1^ and 0.056 cm^3^ g^−1^ of the MnO_2_ raw material to 19.3 m^2^ g^−1^ and 0.049 cm^3^ g^−1^ of the core@shell particles, respectively. These powder property changes are due to the formation of the Li(Ni,Mn)_2_O_4_ particles as the shell. The primary particle size of the MnO_2_ raw material, which was estimated from the specific surface area, was 29 nm. That is, the MnO_2_ raw material consists of the nanometer-sized primary particles. The formation of Li(Ni,Mn)_2_O_4_ particles arises at the nanometer-sized particle of the MnO_2_ surface through mechanical treatment. However, the shearing process of an attrition-type mill scrapes the synthesized Li(Ni,Mn)_2_O_4_ nanoparticles, and the fresh surfaces are created at the MnO_2_ particles. By repeating the formation and abrasion of Li(Ni,Mn)_2_O_4_ particles, the product particles are made more round in shape. Meanwhile, the synthesized Li(Ni,Mn)_2_O_4_ nanoparticles are progressively deposited on the particle surface of MnO_2_, and the core@shell structure is formed during mechanical treatment. This deposition process of the Li(Ni,Mn)_2_O_4_ nanoparticles onto MnO_2_ leads to the increase of the pore diameter and the decrease of the specific surface area. The mechanical treatment using an attrition-type mill allows surface coating of the larger species in the case of an obvious difference in particle size between reactants [30]. In this study, the synthesis of the shell particles and the formation of the core@shell structure were achieved by a one-step dry process.

**Figure 4.**
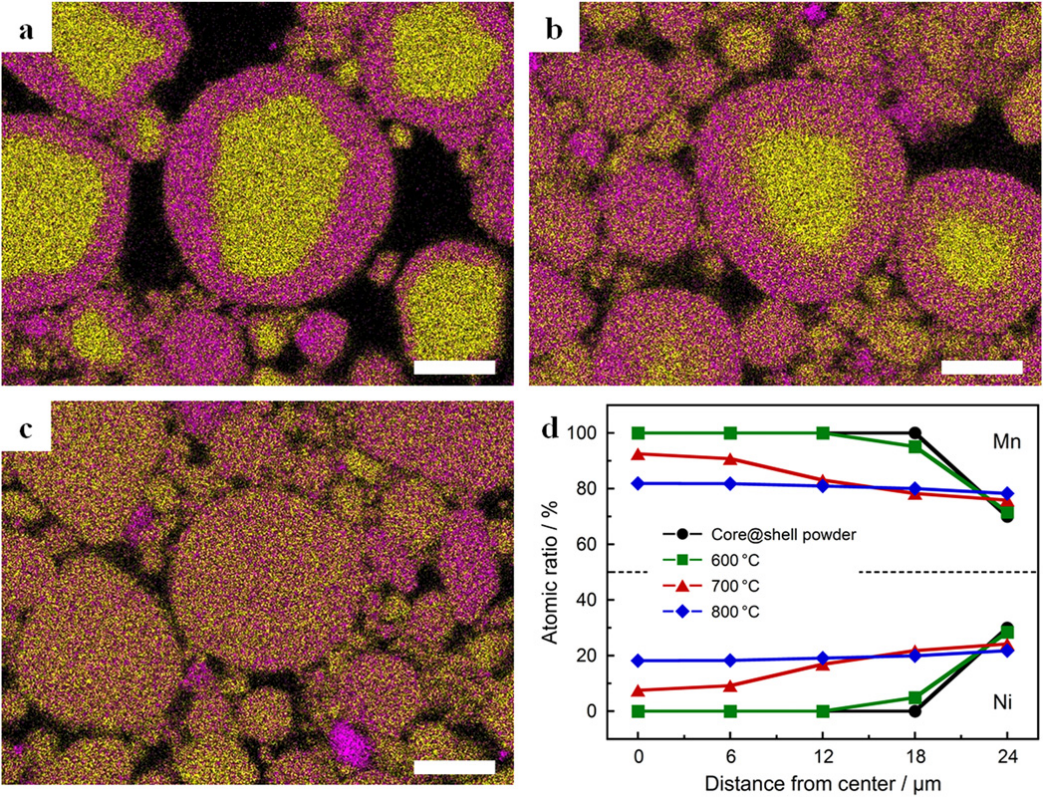
Cross-sectional EDS elemental maps of Ni (purple) and Mn (yellow) for the concentration-gradient powders prepared by calcination at (a) 600 °C, (b) 700 °C and (c) 800 °C for 2 h. Scale bar represents 20 *μ*m. (d) Atomic ratio of transition metals as a function of the distance from the center to the surface for the core@shell and concentration-gradient powders.

The concentration-gradient spinel particles were prepared by calcination of the MnO_2_@Li(Ni,Mn)_2_O_4_ core@shell powder at 600–800 °C for 2 h. The superimposed EDS maps and elemental profiles of the cross-sectional view for the obtained particles are shown in figure 4. The distribution of nickel, which existed in the shell, spread into the core with the increasing calcination temperature (figures 4(a), (b)). After calcination at 800 °C, manganese and nickel were distributed to all the regions of the particles (figure 4(c)). The relative atomic ratio of manganese and nickel from the center to the surface was measured by EDS point analysis, which selected particles with a diameter of ≈50 *μ*m (figure 4(d)). Comparing the atomic ratios in the MnO_2_@Li(Ni,Mn)_2_O_4_ core@shell particle and the concentration-gradient particle obtained by calcination at 600 °C, the nickel ratio slightly increased in the shell part. The product possessing a concentration gradient of manganese and nickel in the entire particle was obtained by calcination at 700 °C. In the case of calcination at 800 °C, the atomic ratio of manganese and nickel was about 80% and 20%, respectively, all through the particle. The formation approaches of the concentration-gradient shell or particle were achieved by changing a calcination temperature of the core@shell particles as a precursor. An elemental analysis of the concentration-gradient particles was conducted by ICP-AES. The total average chemical composition was determined to be LiNi_0.32_Mn_1.68_O_4_ in all the concentration-gradient particles prepared by calcination at 600–800 °C. The doped amount of nickel in this average composition was higher than that in the concentration-gradient spinel particle prepared by Wei *et al* [27].

**Figure 5.**
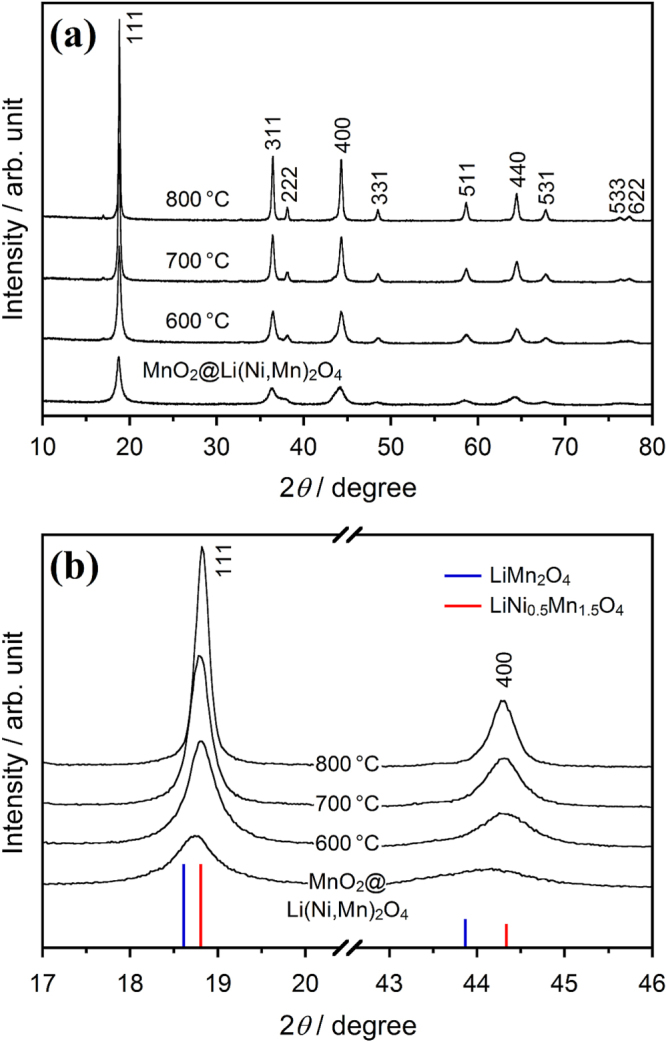
(a) XRD patterns of the MnO_2_@Li(Ni,Mn)_2_O_4_ core@shell powder and the concentration-gradient powders prepared by calcination at various temperatures for 2 h. (b) Expanded XRD patterns for the (111) and (400) peaks of the MnO_2_@Li(Ni,Mn)_2_O_4_ core@shell and concentration-gradient powders. The standard XRD peaks of PDF No. 00-035-0782 for LiMn_2_O_4_ and PDF No. 01-070-8650 for LiNi_0.5_Mn_1.5_O_4_ are also shown.

The XRD patterns of the MnO_2_@Li(Ni,Mn)_2_O_4_ core@shell powder and the concentration-gradient powders prepared by calcination at various temperatures are shown in figure 5. Although a broad XRD pattern was obtained for the core@shell powder, the diffraction peaks were attributed to a cubic spinel structure with a space group of *Fd*-3*m*. This broad XRD pattern is due to the low crystalline MnO_2_ phase of the core and the unreacted NiO phase. By calcination of the core@shell powder, the sharp XRD patterns were obtained, and the separation of (311) and (222) diffraction peaks became clear. A tiny diffraction peak at 17° is due to a *Kβ* peak of the strongest (111) diffraction. The expanded XRD patterns in the 2*θ* regions of 17°–20° and 43°–46° show the (111) and (400) diffraction peaks of their spinel phases, respectively (figure 5(b)). These diffraction peaks for the Li(Ni,Mn)_2_O_4_ shell of the core@shell powder were located between the LiMn_2_O_4_ and LiNi_0.5_Mn_1.5_O_4_ phases as a reference. Consequently, the formation of a Ni-doped LiMn_2_O_4_ phase by mechanical treatment was confirmed by XRD analysis. Both (111) and (400) diffraction peaks shifted to a higher angle by calcination. This peak shift suggests that the MnO_2_ phase of the core decreased with increasing the formation ratio of the spinel phase. The lattice parameter of the concentration-gradient LiNi_0.32_Mn_1.68_O_4_ particles obtained by calcination at 700 °C was calculated by the least square method using silicon as an internal standard material. The calculated lattice parameter of 8.181(1) Å was in agreement with the linear relation estimated from the reported values of the LiNi_0.5−*x*_Mn_1.5+*x*_O_4_ spinels [15].

**Figure 6.**
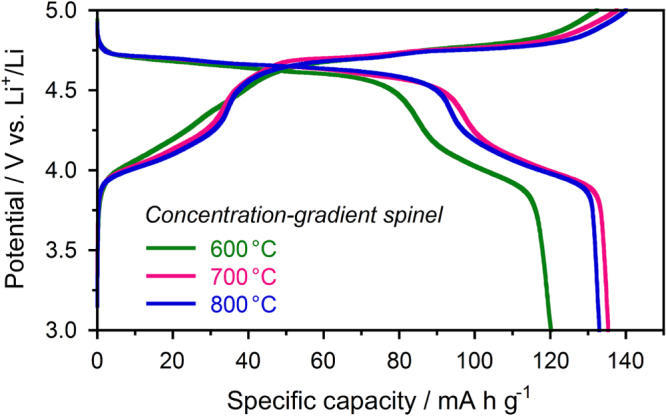
First charge-discharge curves at a constant current of 0.1 C of the concentration-gradient spinel cathodes prepared by calcination at various temperatures.

The electrochemical performances of the concentration-gradient spinel powders were tested using a coin-type half-cell employing Li metal as the anode. Figure 6 shows the first charge-discharge curves of the concentration-gradient spinel cathodes, measuring at a constant current density of 14.6 mA g^−1^ (0.1 C rate for LiNi_0.5_Mn_1.5_O_4_) in a voltage range of 3.0–5.0 V. There are two noteworthy features in our concentration-gradient spinels compared with the reported core@shell and concentration-gradient spinels [21, 27]. One is a high specific discharge capacity. The theoretical values for the LiNi_0.5−*x*_Mn_1.5+*x*_O_4_ (0 ≤ *x* ≤ 0.5) spinels are 146.7–148.2 mA h g^−1^. The concentration-gradient spinel prepared by calcination at 700 °C provided the highest discharge capacity of 135.3 mA h g^−1^, whereas that at 600 °C was 120.2 mA h g^−1^. The low discharge capacity at 600 °C is caused by a remaining MnO_2_ phase. This high capacity will lead to an increase of the energy density on the practical applications. The other feature is the presence of an electrochemically active region at a high voltage of 4.6–4.7 V. A capacity of about 90 mA h g^−1^ was delivered by this high-voltage region. The operation voltages of LiMn_2_O_4_ and LiNi_0.5_Mn_1.5_O_4_ spinels are 4.1 V and 4.7 V, which are attributed to the redox couples of Mn^3+^/Mn^4+^ and Ni^2+^/Ni^4+^, respectively [9, 15]. Therefore, the concentration-gradient LiNi_0.32_Mn_1.68_O_4_ spinels had a close property to the LiNi_0.5_Mn_1.5_O_4_ spinel.

**Figure 7.**
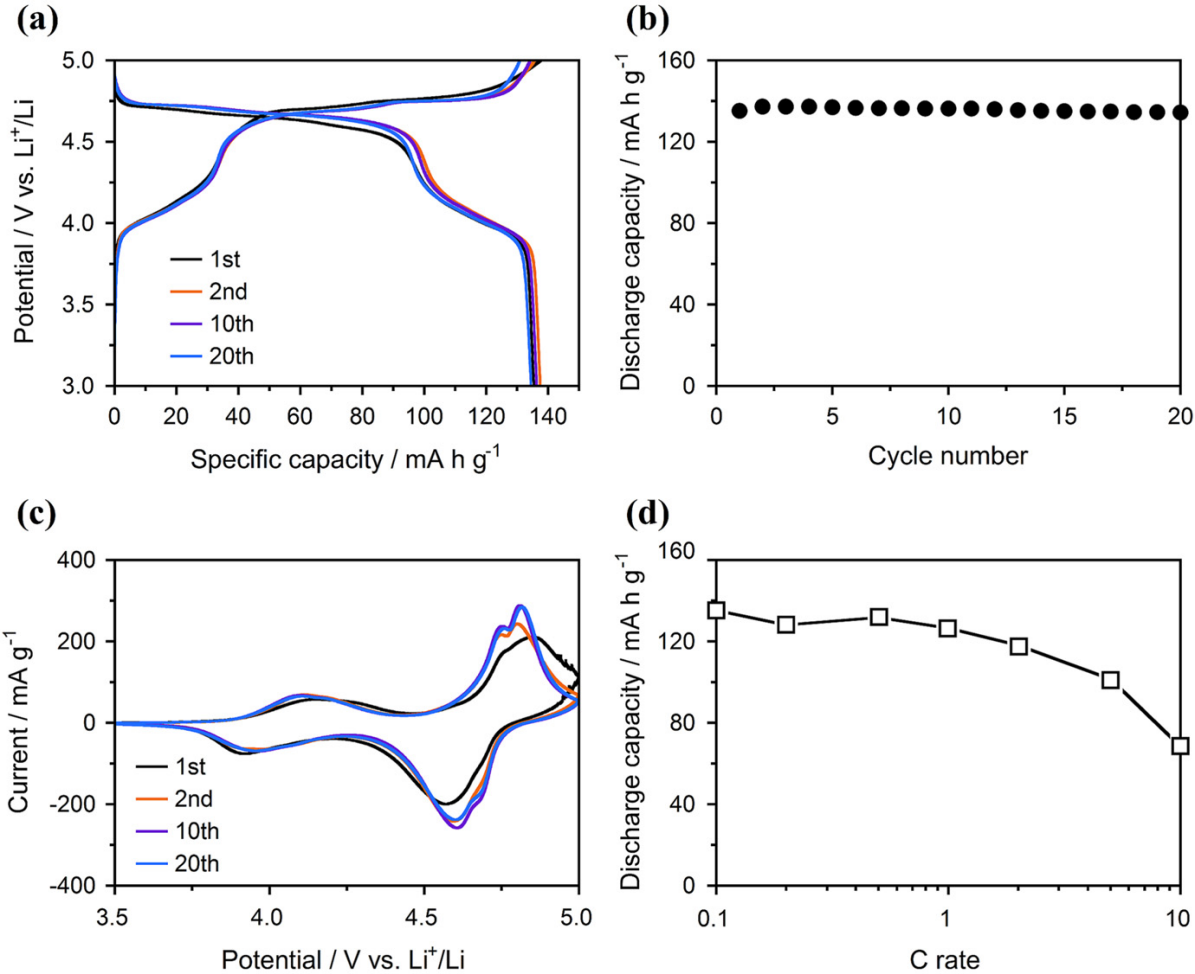
Initial electrochemical performances of the concentration-gradient LiNi_0.32_Mn_1.68_O_4_ spinel cathode prepared by calcination at 700 °C: (a) charge-discharge curves and (b) cycle performances at a constant current of 0.1 C, (c) CV curves at a scan rate of 0.2 mV s^−1^ and (d) initial discharge capacities at different current rates.

From the results of the first charge-discharge curves, the concentration-gradient LiNi_0.32_Mn_1.68_O_4_ spinel prepared by calcination at 700 °C was selected to evaluate the further electrochemical performances. Although the obvious difference between the spinel cathodes prepared at 700 °C and 800 °C were not found in the first charge-discharge curves, calcination at a high temperature may lead to the formation of a Li_1−*x*_Ni_*x*_O phase [15]. The cycle behavior and cyclic voltammograms (CVs) during the initial 20 cycles and rate properties are summarized in figure 7. There was no change in the charge-discharge curves during 20 cycles at a constant current density of 14.6 mA g^−1^ (figure 7(a)). Accordingly, the discharge capacities during the cycles were recorded at around 135 mA h g^−1^ (figure 7(b)). The capacity retention after 20 cycles was 99.4%. If the discharge capacity decreases with increasing a cycle number, the capacity retention after 100 cycles extrapolated from the 20 cycles data shows 91.0%. However, the degradation by oxidation of an electrolyte at a high-voltage region is not negligible [31, 32], so it is necessary to further increase the cycle number. The CV curves recorded at a scan rate of 0.2 mV s^−1^ indicated the three redox peaks (figure 7(c)). A small hump centered at about 4.1 V corresponds to the redox couple of Mn^3+^/Mn^4+^, which is referred from the Mn^3+^ constituent in the concentration-gradient LiNi_0.32_Mn_1.68_O_4_ spinel. The partially overlapped peaks at about 4.7 and 4.8 V correspond to the redox couples of Ni^2+^/Ni^3+^ and Ni^3+^/Ni^4+^, respectively. These redox couples showed the reversibility and the cyclability. The rate properties of the concentration-gradient LiNi_0.32_Mn_1.68_O_4_ spinel cathode were tested up to 1.46 A g^−1^ (10 C rate) (figure 7(d)). The charge rate was equal to the discharge rate, and the constant-voltage charge for 5 h was conducted after achieving maximum voltage of 5 V. The first discharge capacities over 125 mA h g^−1^ were recorded until reaching a 1 C rate. Although the discharge capacity gradually decreased with increasing the applied current densities, 68.4 mA h g^−1^ was yielded at a 10 C rate. The discharge capacities at each rate were almost the same after 3 cycles. The concentration-gradient LiNi_0.32_Mn_1.68_O_4_ spinel showed favorable cathode properties in the initial electrochemical performances.

## 4. Conclusions

We have demonstrated a facile method to prepare the core@shell and concentration-gradient spinel particles. The preparation method of the concentration-gradient spinels with an average composition of LiNi_0.32_Mn_1.68_O_4_ consists of the mechanical synthesis of the MnO_2_@Li(Ni,Mn)_2_O_4_ core@shell particles using an attrition-type mill and the calcination step of those as a precursor. The core@shell and concentration-gradient structures were confirmed by EDS elemental maps and profiles. According to the cell tests using a coin-type half-cell employing Li metal as the anode, the concentration-gradient LiNi_0.32_Mn_1.68_O_4_ spinel cathode prepared by calcination at 700 °C exhibited the high discharge capacity of 135.3 mA h g^−1^, the wide-range plateau at a high voltage of 4.7 V and the cyclability with a capacity retention of 99.4% after 20 cycles. These concentration-gradient LiNi_0.32_Mn_1.68_O_4_ spinel particles are expected to be used as a cathode for high-energy LIBs, though a further examination of its cyclability is necessary. Additionally, the facile preparation route of the core@shell and concentration-gradient particles demonstrated in this study may provide a new opportunity for the discovery and investigation of functional materials as well as for the cathode materials for LIBs.
